# The Influence of the Third Element on Nano-Mechanical Properties of Iron Borides FeB and Fe_2_B Formed in Fe-B-X (X = C, Cr, Mn, V, W, Mn + V) Alloys

**DOI:** 10.3390/ma13184155

**Published:** 2020-09-18

**Authors:** Ivana Kirkovska, Viera Homolová, Ivan Petryshynets, Tamás Csanádi

**Affiliations:** 1 Institute of Materials Research, Slovak Academy of Sciences, Watsonova 47, 040 01 Kosice, Slovakia; vhomolova@saske.sk (V.H.); ipetryshynets@saske.sk (I.P.); tcsanadi@saske.sk (T.C.); 2 Faculty of Materials, Metallurgy and Recycling, Technical University of Košice, Letná 9, 042 00 Košice, Slovakia

**Keywords:** iron borides, hardness, modulus, nanoindentation, indentation size effect, Fe-B-X (X = C, Cr, Mn, V, W, V + Mn) alloys

## Abstract

In this study, the influence of alloying elements on the mechanical properties of iron borides FeB and Fe_2_B formed in Fe-B-X (X = C, Cr, Mn, V, W, Mn + V) alloys were evaluated using instrumented indentation measurement. The microstructural characterization of the alloys was performed by means of X-ray diffraction and scanning electron microscope equipped with an energy dispersive X-ray analyzer. The fraction of the phases present in the alloys was determined either by the lever rule or by image analysis. The hardest and stiffest FeB formed in Fe-B-X (X = C, Cr, Mn) alloys was observed in the Fe-B-Cr alloys, where indentation hardness of H_IT_ = 26.9 ± 1.4 GPa and indentation modulus of E_IT_ = 486 ± 22 GPa were determined. The highest hardness of Fe_2_B was determined in the presence of tungsten as an alloying element, H_IT_ = 20.8 ± 0.9 GPa. The lowest indentation hardness is measured in manganese alloyed FeB and Fe_2_B. In both FeB and Fe_2_B, an indentation size effect was observed, showing a decrease of hardness with increasing indentation depth.

## 1. Introduction

Owning to their characteristics, i.e., high hardness, thermal stability, high wear and corrosion resistance, metal borides are considered a perspective option where the operational conditions demand for improved performance, reliability, safety and increased service life of various engineering components [1]. For example, the use of metal borides as protective coatings is one technological area of considerable practical importance in the case of both ferrous and non-ferrous alloys [2]. The surface modification of the materials is achieved by formation of a dual layer FeB + Fe_2_B or a monophase Fe_2_B layer by high-temperature diffusion of boron atoms (boronizing process) [3]. As such, these materials are utilized, for example, in the production of forging and stamping dies used in the manufacturing industry or valves, bearings and other production tubing system components used in the oil and gas industry [4,5]. Equally important is the application of boron for hardness enhancement through alloying, where the borides formed are utilized as hard reinforcing phases, e.g., boron alloyed tool steels [6,7], and high-boron white cast irons [8,9,10,11,12], etc. 

Numerous information on FeB and Fe_2_B borides can be obtained from boronizing processes research, where these borides have been of primary interest. Available studies on mechanical and tribological properties of boride layers vouch for their advantages over other surface hardening treatments [13,14,15,16]. Reported microhardness values for boride layers are mainly in the range between 1600 and 2200 HV_0.05_ [17,18,19]. The wide range in reported values can be due to differences in characterization technique used, but even more so is a result of the different alloying elements present in the substrate material [20,21,22,23]. 

Over recent years, a number of studies on nanomechanical characterization of FeB and/or Fe_2_B iron borides produced by boronizing have been published [24,25,26,27,28]. The difference among the reported values obtained by nanomechanical characterization are often prescribed to mixed phase effect, porosity, residual stresses, and differences in chemical composition of the substrate material [29]. In order to eliminate the substrate influence, Kulka et al. [27] conducted a boronizing study on pure iron (Armco iron). Berkovich nanoindentation measurement resulted in the following hardness values: FeB = 19.77–29.35 GPa and Fe_2_B = 16.09–18.51 GPa [27], and indentation modulus of FeB = 271.89–360.16 GPa and Fe_2_B = 231.52–275.37 GPa [27]. 

Recent studies identified metal borides, and particularly Fe_2_B, as potential strengthening phases [29,30,31,32]. However, limited experimental work is available on the nanomechanical properties of Fe_2_B, and even more so on FeB phase, as precipitate phases in boron alloyed materials [29,33,34]. In addition, lately, there is interest across scientific groups on studying the effects of alloying on the mechanical properties of hard boride phases [35,36,37]. The research has shown that the properties can be significantly influenced by the alloying elements, but still their individual influence has not been well defined and entirely understood. 

Hence, the primary objective of this study is to investigate the nanomechanical properties like indentation hardness (H_IT_) and indentation modulus (E_IT_) of the FeB (MB) and Fe_2_B (M_2_B) phases, as precipitate phases. Furthermore, examination of the nanomechanical properties of precipitates FeB and Fe_2_B phases with respect to indentation size effect (ISE) for selected alloys is provided. The investigative Fe-B alloys of the following type: B-Fe-C, B-Fe-Cr, B-Fe-Mn, B-Fe-V, and B-Fe-W, were chosen as such, so as to be able to isolate as much as possible the effect of the third compositional element present in the iron borides on their nanomechanical properties. In general, the solubility of tungsten (W) and carbon (C) in iron borides is low, while the solubility of chromium (Cr) reaches up to 15 at.% for Fe_2_B phase, and 16 at.% for FeB boride at 1353 K [38]. Manganese (Mn) can completely replace iron in these borides [39], therefore it is interesting to study the influence of these elements on borides mechanical properties. One equilibrated quaternary Fe-B-Mn-V alloy was also used in order to assess the combined effect of Mn and V as alloying elements. It should be noted that due to the complete mutual solubility between compounds MnB and FeB, and Mn_2_B and Fe_2_B, notations MB and M_2_B are used for these phases formed in Fe-B-Mn alloys. M denotes metallic element also in the higher order system Fe-B-Mn-V.

## 2. Materials and Methods 

The Fe-B-X alloys were prepared from high-purity powders (Fe-99.98 % or 99.96 %, B-99.95 %, C-99.90 %, Cr-99.99%, Mn-99.98 % or 99.95 %, V-99.80 %, W-99.90 %). Details on the production process can be found in published studies on phase equilibria and/or thermodynamic modelling studies [38,39,40,41,42,43,44,45,46]. The mixed powders were pressed into cylindric compacts using a uniaxial pressing machine. The subsequent melting was done using induction melting and argon arc melting in the case of Fe-B-C alloys. Fe-B-X (X = Cr, Mn, V, W, Mn + V) alloys were melted in an argon arc furnace (Mini Arc Melter MAM-1). The alloys were melted several times in order to ensure homogeneity. The produced alloys weighted 10–20 g in the case of Fe-B-C, 15 g for Fe-B-Cr, 5–7 g for Fe-B-Mn alloys, 10–20 g for Fe-B-V alloys, 2 g and 3. 5 g alloys in the case of the Fe-B-W system, and 7 g for Fe-B-Mn-V alloy. Further, the as-cast alloys were evacuated in silica glass tubes and long-term annealed using electric resistance furnace LAC-type L 06 S. For oxidation elimination, titanium turnings were placed into the silica tubes. Following the annealing treatment, the alloys were quenched into cold water. Alloy designation, chemical composition, and annealing conditions are given in Table 1. Afterward, the produced alloys were sectioned in half using electro-sparking and a metallographic procedure following a sequence of mechanical grinding, and polishing (using 120 up to 4000 grit SiC paper) was applied in order to prepare the alloys for analysis. The microstructure and the chemical composition of the phases were analyzed using a FEG SEM facility JEOL JSM-7000F equipped with an INCA EDX analyzer. The micrographs were taken in backscatter imaging mode at 10 kV acceleration voltage. The phase composition of the alloys was determined by X-ray diffraction (XRD) using Philips X’Pert Pro MPD with a Bragg–Brentano setup or Bruker D8 Advance diffractometer (Bruker, USA) in Bragg–Brentano pseudofocusing geometry (in the case of the Fe-B-V-Mn alloy). Cu Kα and Cr Kα (only for Fe-B-Cr alloys) radiation with wavelength λ = 1.540562 Å and λ = 2.289700 Å was used in the diffraction experiments. Detailed X-ray diffraction results can be found in previously published studies on phase equilibria and/or thermodynamic modeling studies [38,39,42,43,44,45].

The volume fraction of the identified phases in alloys Fe-B-C, Fe-B-Cr, Fe-B-W, and Fe-B-Mn-V was determined by image analysis. The volume fraction was derived from the area fraction. Namely, the area fraction can be considered as equivalent to the volume fraction under the assumption of homogenous and isotropic materials. Image analysis was done using the open-source scientific processing program ImageJ/Fiji [47]. The volume fraction of the identified phases present in Fe-B-Mn and Fe-B-V alloys was calculated using the lever rule. For this purpose, experimentally determined chemical composition values for the phases of interest and the overall composition of the alloys were used as input data, followed by conversion from mole percent to volume percent. Information on phases’ molar volume are taken from Repovský et al. [39]. The determined volume fractions are given in Table 2.

For the nanoindentation testing, the sectioned alloys were mounted using the compression thermosetting molding technique, and then they were ground and polished—flat. In addition, a final automatic polishing step was used to ensure a smooth top surface. Using crystal-bond hot-melt thermoplastic polymer, the alloys were mounted on aluminum sample disks, and then installed into a sample holder. Nanoindentation experiments were performed using Nano Indenter G200 produced by MTS Nano Instruments equipped with a Berkovich-type diamond indenter. Poisson’s ratio (ν) of 0.3 is used, for both FeB (MB) and Fe_2_B (M_2_B) phases, assuming a quasi-isotropic behavior. Measurements were done using both single loading–unloading indentation and continuous stiffness measurement (CSM) methods. The latter was applied to study the indentation size effect (ISE). 

In both cases, an indentation depth-controlled method was used with a maximum depth of 500 nm. On average, 25 indentation tests were carried out on FeB and/or Fe_2_B phase for determining the indentation hardness and modulus of the alloys. In addition, 10 depth-controlled indentation tests to a maximum depth of 500 nm were performed with an aim to inspect for the presence of indentation size effect (ISE). The area function of the indenter tip was calibrated using fused silica preceding the indentation tests.

## 3. Results and Discussion

### 3.1. Phase Composition and Microstructure 

**Figure 1:**
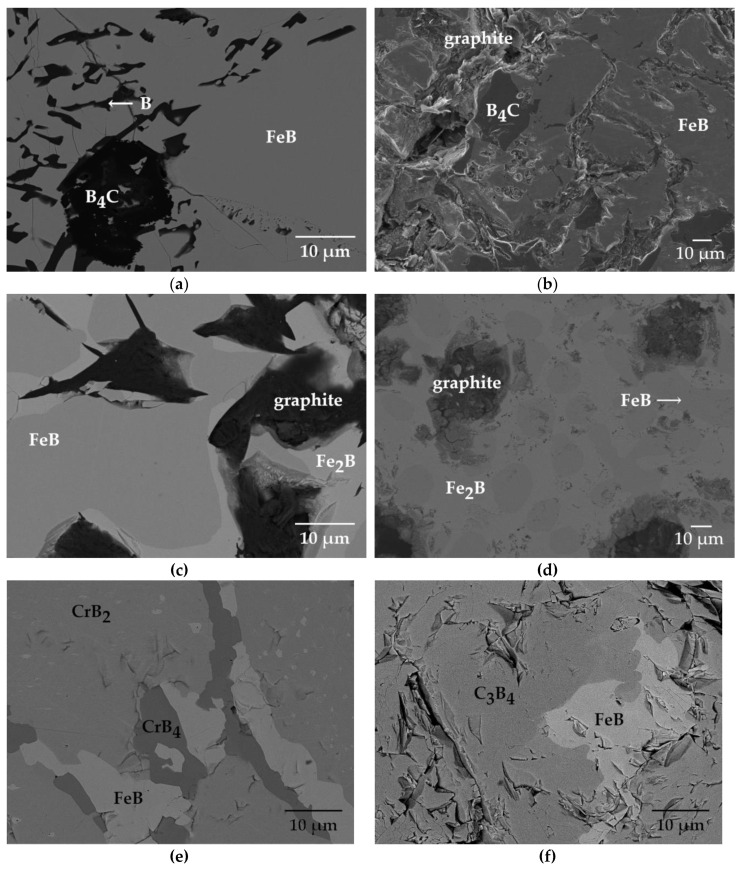

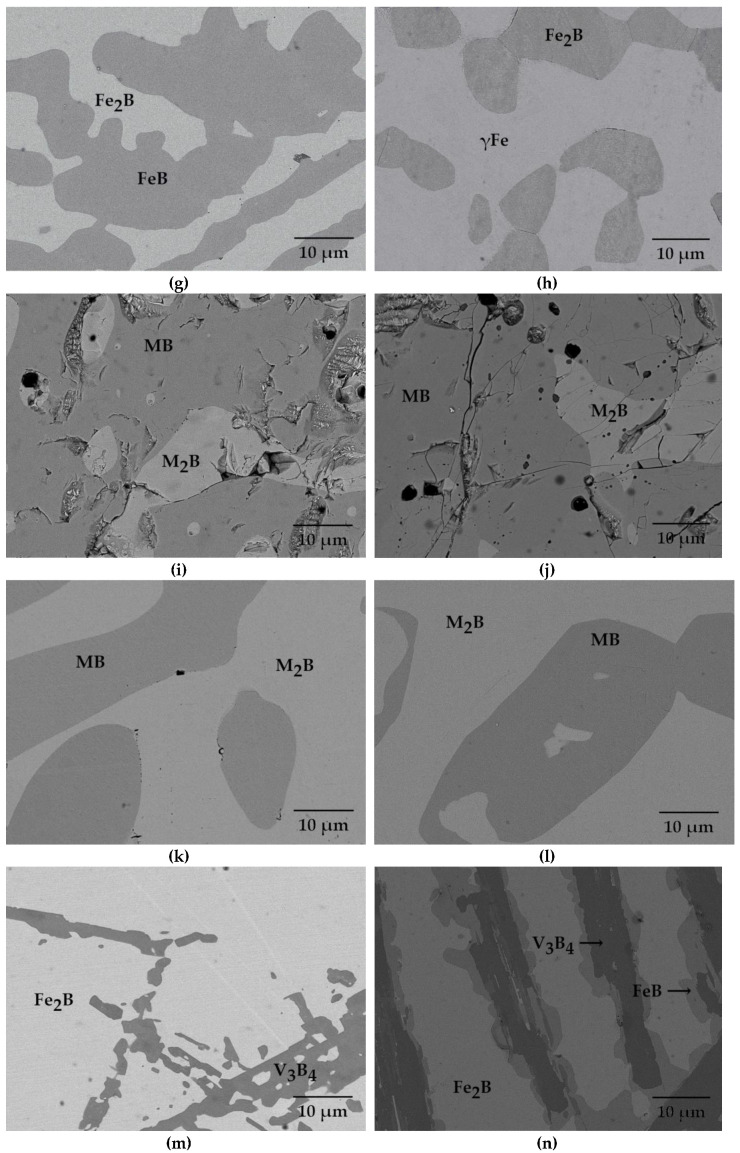

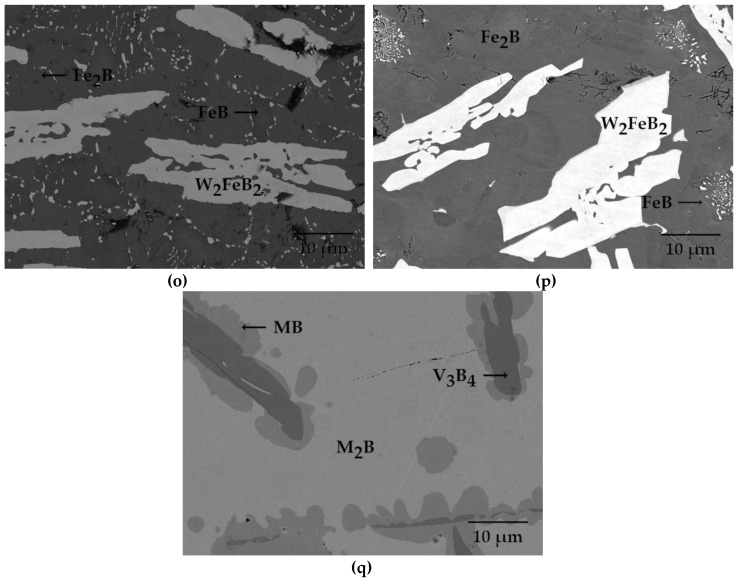
Alloys’ micrographs (**a**) 38.5 Fe–59.22 B–2.3 C; (**b**) 34.6 Fe–52 B–13.4 C; (**c**) 37 Fe–34 B–29 C; (**d**) 39.7 Fe–33 B–27.3 C; (**e**) 17 Fe–65 B–18 Cr; (**f**) 8 Fe–56 B–36 Cr; (**g**) 50 Fe–40 B–10 Cr; (**h**) 82 Fe–9 B–9 Mn; (**i**) 22 Fe–39 B–39 Mn (alloy 9); (**j**) 22 Fe–39 B–39 Mn (alloy 10); (**k**) 51 Fe–39 B–10 Mn (alloy 11); (**l**) 51 Fe–39 B–10 Mn (alloy 12); (**m**) 50 Fe–41 B–9 V (alloy 13); (**n**) 50 Fe–41 B–9 V (alloy 14); (**o**) 51 Fe–42 B–7 W (alloy 15); (**p**) 51 Fe–42 B–7 W (alloy 16); (**q**) 45 Fe–40 B–5 Mn–10 V.

Sixteen equilibrated ternary Fe-B-X (X = C, Cr, Mn, V, W) alloys and one equilibrated quaternary Fe-B-Mn-V alloy were investigated in this study. The microstructures of the investigated alloys are shown in Figure 1. Alloys’ phase composition is given in Table 1. 

Two-phase microstructure was identifiable in eight of the investigated ternary alloys. The rest of the ternary alloys were characterized by three-phase microstructure. Three-phase microstructure is also observed in the quaternary Fe-B-Mn-V alloy. In the majority of alloys, both FeB (MB) and Fe_2_B (M_2_B) are present (Figure 1). Exceptions make the following alloys: 38.5 Fe–59.22 B–2.3 C, 34.6 Fe–52 B–13.4 C, 17 Fe–65 B–18 Cr, and 8 Fe–56 B–36 Cr, where only FeB is found (Figure 1a,b,e,f). Whilst, in alloys 82 Fe–9 B–9 Mn and 50 Fe–41 B–9 V (alloy 13), only Fe_2_B is observed (Figure 1h,m).

Although, FeB and Fe_2_B phases are present in various shapes and dimensions, still, in most of the alloys, phases of 10 μm in diameter are easily identifiable (Figure 1). Iron borides relevant crystallographic structure information are as follows: FeB (Pearson symbol—*oP*8, Proto-type FeB, Space group *Pnma*) and Fe_2_B (Pearson symbol—*tI*12, Proto-type CuAl_2_, Space group—*I*4/*mcm*) [48]. 

The determined phase fractions of the investigated alloys are shown in Table 2. Values calculated by image analysis are consistent with location of the individual alloys in phase equilibrium regions of the corresponding phase diagrams. The lever rule method is essentially based on the location of the alloy in the phase equilibrium fields, so that the values calculated by this method are, of course, in accordance with it.

The iron borides dissolve the third element (in case of the quaternary alloy, also the fourth element) in greater or lesser amounts in all investigated alloys. The amount of the third element dissolved in FeB and Fe_2_B phases in the investigated ternary alloys and the dissolved amount of vanadium and manganese in iron borides in investigated quaternary alloy are given in Table 1.

#### 3.1.1. Fe-B-C Alloys 

The FeB phase is present in each of the investigated alloys of the Fe-B-C type (Figure 1a–d). The FeB phase is the predominant phase both in phase diameter and volume fraction in the microstructure of 38.5 Fe–59.2 B–2.3 C, 34.6 Fe–52 B–13.4 C, and 37 Fe–34 B–29 C alloys (Table 2, Figure 1a–c). The Fe_2_B phase is present in only two of the Fe–B–C alloys, i.e., 37 Fe–34 B–29 C and 39.7 Fe–33 B–27.3 C (Figure 1c,d). The B_4_C phase is identified in the microstructure of alloys 38.5 Fe–59.22 B–2.3 C and 34.6 Fe–52 B–13.4 C in small portions (Table 2). However, it should be noted that the given phase fraction for graphite, when present in the alloy, is overestimated at an expanse of B_4_C, Fe_2_B, and FeB phases, since it is very difficult to distinguish the border between graphite and these other phases. 

#### 3.1.2. Fe-B-Cr Alloys 

FeB is identified in the microstructure of each of the Fe-B-Cr alloys (Figure 1e–g). In alloys 17 Fe–65 B–18 Cr and 8 Fe–56 B–36 Cr, the FeB is surrounded by chromium borides CrB_4_, CrB_2_, and Cr_3_B_4_, respectively (Figure 1e,f). The microstructure of alloy 17 Fe–65 B–18 Cr mostly consists of Fe_2_B, i.e., 71.35 vol.%, while in the 8 Fe–56 B–36 Cr alloy, C_3_B_4_ phase occupies the biggest portion of the microstructure, with 97.13 vol.%. (Table 2). In alloy 50 Fe–40 B–10 Cr, the FeB phase (dark gray) is present, together with light gray Fe_2_B phase, each characterized with relatively high chromium solubility of 8 at.% and 16 at.%, respectively (Table 1, Figure 1g). 

#### 3.1.3. Fe-B-Mn Alloys 

With exception to the 82 Fe–9 B–9 Mn alloy, each of the Fe-B-Mn alloys are comprised of FeB (MB) and Fe_2_B (M_2_B) phases (Table 1, Figure 1h–l). The Fe-B-Mn ternary system is characterized by complete mutual solubility of Fe_2_B and Mn_2_B, and FeB and MnB phases [39]. Iron (Fe) took over the greatest proportion of metallic elements in all borides in the Mn alloys under study here, with exception to 22 Fe–39 B–39 Mn (alloy 9) and 22 Fe–39 B–39 Mn (alloy 10), where manganese (Mn) was the dominant element (Table 1). Additional information for Fe-B-Mn alloys are given in the studies by Repovsky et al. [39] and Kirkovska et al. [49]. 

#### 3.1.4. Fe-B-V Alloys 

Two Fe-B-V alloys were investigated in this study, alloy 13 (50 Fe–41 B–9 V annealing conditions 1353 K/1440 h) and alloy 14 (50 Fe–41 B–9 V annealing conditions 903 K/4560 h) (Figure 1m,n). The phase fraction analysis shows that Fe_2_B phase takes up the biggest portion of the microstructure, 76.7% and 63.8%, for alloys 13 and 14, respectively (Table 2). In alloy 13, the large dark grey plates of V_3_B_4_ are embedded in light gray Fe_2_B matrix (Figure 1m). The dendrite-like structure identified as V_3_B_4_ (darkest color) is surrounded by FeB phase (medium color), embedded in the (light gray) Fe_2_B matrix in alloy 14 (Figure 1n). 

#### 3.1.5. Fe-B-W Alloys 

**Figure 2:**
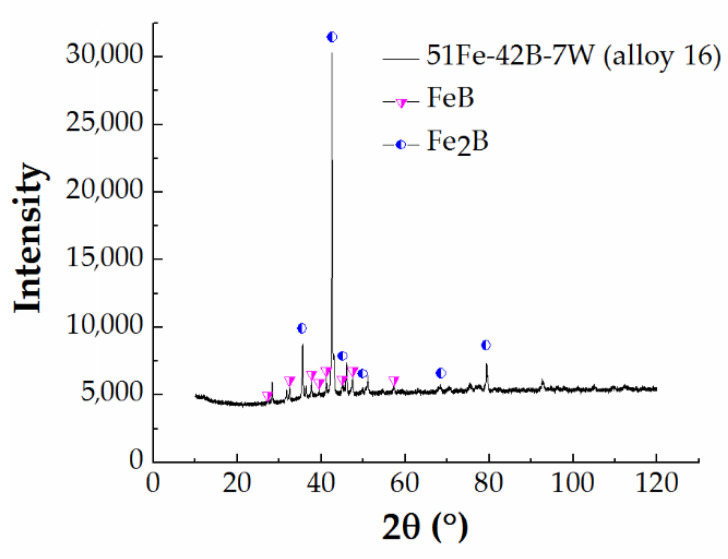
Diffraction pattern of 51 Fe–42 B–7 W (alloy 16) with identified binary FeB and Fe_2_B phases.

Two Fe-B-W alloys (Figure 1o,p) were investigated in the present study. X-ray analysis results confirm the existence of FeB and Fe_2_B (Figure 2). The experimentally determined equilibrium composition of the phases present in these alloys, using energy-dispersive X-ray spectroscopy (EDX/EDS) coupled with published Fe-B-W phase diagram studies [48], indicate the existence of W_2_FeB_2_ for the unidentified picks. Hence, a three-phase microstructure consisting of FeB + Fe_2_B + W_2_FeB_2_ was identified. With long-term annealing at the higher temperature (1323 K), the FeB phase appears more refined (Figure 1o,p). Also, with long-term annealing, the phase fraction of W_2_FeB_2_ ternary boride increases slightly, alongside FeB phase, at the expense of Fe_2_B phase (Table 2).

#### 3.1.6. Fe-B-Mn-V Alloys 

**Figure 3:**
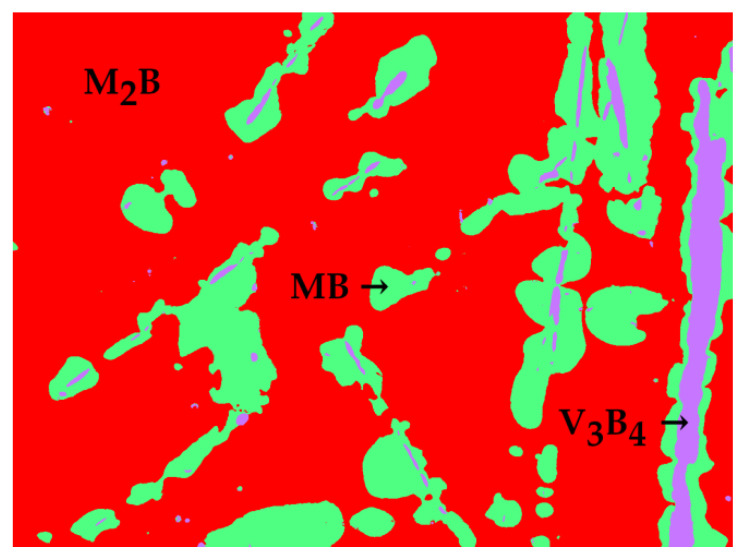
Segmented image of the microstructure of alloy 45 Fe–40 B–5 Mn–10 V (alloy 17).

M_2_B, MB, and V_3_B_4_ are identified in the microstructure of the Fe-B-Mn-V alloy (Figure 1q). The matrix consists of light gray M_2_B phase. The dark gray phase embedded in the matrix is identified as V_3_B_4_. V_3_B_4_ is surrounded by MB phase. Here, vanadium and manganese are both dissolved in iron borides M_2_B and MB. Additional information about Fe-B-Mn-V quaternary can be obtained from Homolová et al. [42]. A segmented image of the microstructure of alloy 17 used for phase fraction calculation is given in Figure 3. The different colored segments are obtained using the image analysis technique and represent different phases, as labeled in Figure 3. The M_2_B phase is the predominant phase, occupying 71.28 vol.% of the Fe-B-Mn-V alloy microstructure (Table 2).

### 3.2. Hardness and Modulus of FeB and Fe_2_B Borides 

**Figure 4:**
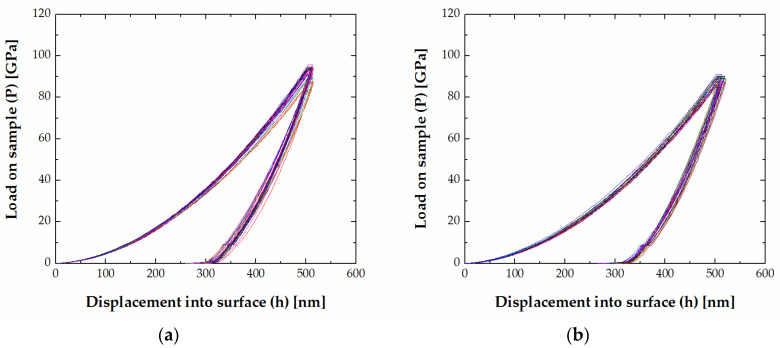
Load vs. depth curves (**a**) FeB phase in alloy 38.5 Fe–59.2 B–2.3 C; (**b**) Fe_2_B phase in 50 Fe–41 B–9 V (alloy 14).

The average hardness and indentation modulus of both FeB and Fe_2_B phase are given in Table 3. The averaged hardness values of FeB and Fe_2_B are in the range of 18.6 ± 0.6–26.9 ± 1.4 GPa and 16.1 ± 2.2–20.8 ± 0.9 GPa, respectively. The lowest hardness and modulus value of the Fe_2_B phase is measured in manganese-type alloy 82 Fe–9 B–9 Mn, H_IT_ (Fe_2_B) = 16.1 ± 2.2 GPa and E_IT_ (Fe_2_B) = 297.5 ± 24.6 GPa. The highest hardness of the Fe_2_B phase is measured in the alloy with tungsten 51 Fe–42 B–7 W, H_IT_ (Fe_2_B) = 20.8 ± 0.9 GPa and highest modulus of Fe_2_B phase in 50 Fe–40 B–10 Cr, a chromium-containing alloy, E_IT_ (Fe_2_B) = 391.8 ± 10.9 GPa. The highest and lowest hardness of the FeB is measured in the alloy with chromium 17 Fe–65 B–18 Cr, H_IT_ (FeB) = 26.9 ± 1.4 GPa and manganese-containing alloy 22 Fe–39 B–39 Mn (alloy 10), H_IT_ (FeB) = 18.6 ± 0.6. E_IT_ = 315.6 ± 32.8 measured in 37 Fe–34 B–29 C, a carbon-type alloy, is the lowest indentation modulus determined for the FeB phase, and E_IT_ = 485.5 ± 22.3 measured in 17 Fe–65 B–18 Cr chromium-type alloy is the highest value of indentation modulus determined for the FeB phase. The load vs. depth (P–h) plots obtained from the nanoindentation experiments on FeB phase in 38.5 Fe–59.2 B–2.3 C alloy and Fe_2_B phase in alloy 50 Fe–41 B–9 V (alloy 14) are shown in Figure 4. 

Comparative evaluation of the results for these alloys show that Cr alloyed FeB is the hardest and stiffest boride. W alloyed Fe_2_B is observed as the hardest boride in the Fe_2_B group. Overall, the hardness is lowest in the Mn alloyed FeB and Fe_2_B. For these alloys, the alloying elements have more influence on the hardness of FeB than on Fe_2_B phase. The indentation hardness and indentation modulus for Fe_2_B distinguished according to the alloying element present, determined in this study, are consistent with reported values for similar borides [33,35,50]. However, there are discrepancies between the reported valued of hardness and modulus in the literature, alongside disagreement of the effect of the alloying elements in terms of decrease/increase of hardness/modulus of Fe_2_B [35,50,51,52,53,54]. We were not able to obtain any information on the influence of alloying elements on mechanical properties of precipitated FeB phase, or information on the effect of alloying additions on FeB from first—principle, studies.

**Figure 5:**
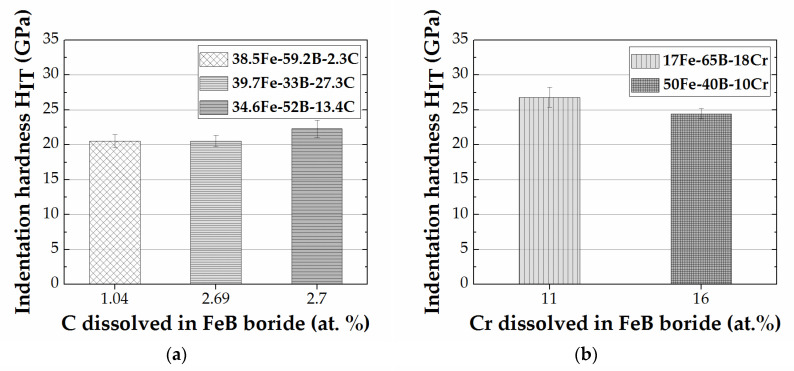

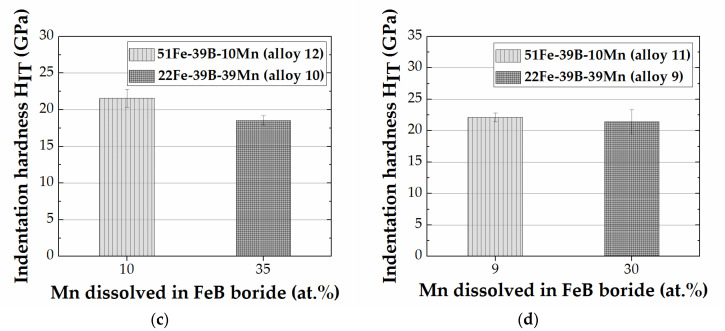
(**a**) Indentation hardness of FeB phase as a function of amount of C dissolved, for Fe-B-C alloys at 1173 K. (**b**) Indentation hardness of FeB phase as a function of amount of Cr dissolved, for Fe-B-Cr alloys at 1353 K. (**c**) Indentation hardness of FeB phase as a function of amount of Mn dissolved, for Fe-B-Mn alloys at 1223 K. (**d**) Indentation hardness of FeB phase as a function of amount of Mn dissolved in this boride in Fe-B-Mn alloys at 873 K.

**Figure 6:**
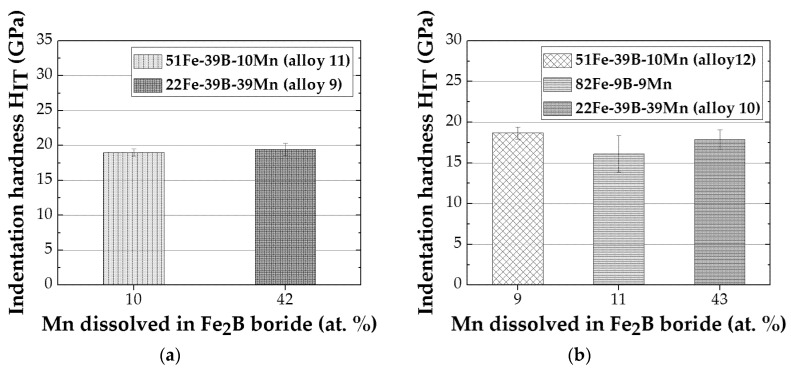
(**a**) Indentation hardness of Fe_2_B phase as a function of the amount of Mn dissolved for Fe-B-Mn alloys at 873 K. (**b**) Indentation hardness of Fe_2_B phase as a function of the amount of Mn dissolved for Fe-B-Mn alloys at 1223 K.

For the FeB (MB) formed in the Fe-B-C, Fe-B-Cr, and Fe-B–Mn systems, the indentation modulus and hardness are determined as a function of the amount of the third element dissolved for specific annealing conditions. The FeB (MB) indentation hardness in the Fe-B-C, Fe-B-Cr, and Fe-B-Mn systems as a function of carbon (C), chromium (Cr), and manganese (Mn) are given in Figure 5. The annealing conditions, i.e., temperature/time (Table 1) allow for comparison of the M_2_B only in alloy system Fe-B-Mn. Thus, the indentation hardness for the M_2_B in Fe-B-Mn alloys heat-treated at 1223 K and 873 K are determined as a function of the amount of manganese dissolved (Mn), and are given in Figure 6.

For the FeB formed in Fe-B-C alloys (Figure 5a), the difference in indentation hardness between alloy 38.5 Fe–59.2 B–2.3 C (1.04% C) and alloy 39.7 Fe–33 B–27.3 C (2.69% C) can be considered negligible. A sharp increase in the measured indentation hardness, i.e., 22.3 ± 1.3 GPa (Figure 4a), with a further increase in carbon content (2.7 at.%) in alloy 34.6 Fe–52 B–13.4 C was observed. The indentation hardness measured cannot be only associated with the C content variation in the alloy. Extrinsic influence in alloy 39.7 Fe–33 B–27.3 C (2.69% C) caused by the surrounding Fe_2_B can lead to underestimation of the indentation hardness. In addition, the high-volume percent of FeB in alloy 34.6 Fe–52 B–13.4 C, i.e., 54.25 vol.%, can be the reason for this difference. The indentation hardness in Cr alloyed FeB is higher at lower amounts of dissolved chromium (Figure 5b). In a like manner, the indentation hardness of FeB containing manganese is higher at lower amounts of dissolved manganese (Figure 5c,d). FeB indentation modulus shows the same behavior as the indentation hardness for the Fe-B-Cr and Fe-B-Mn alloys.

For the manganese alloyed Fe_2_B annealed at 873 K, only a slight increase in hardness is observed with higher amounts of manganese dissolved (Figure 6a). For the Fe_2_B formed in Fe-B-Mn alloys annealed at 1223 K, the decrease in hardness at 11 at.% Mn dissolved is followed by a hardness increase at 43 at.% Mn dissolved (Figure 6b). Fe_2_B indentation modulus for the Fe-B-Mn alloys annealed at 1223 K shows the same behavior. For the Fe_2_B in Fe-B-Mn alloys annealed at 873 K, the indentation modulus is lower at higher manganese content. Nonetheless, when probing phases during nanoindentation testing as a part of a multiphase material, it must be accounted for the possible influences of the surrounding phases/matrix. The extrinsic influences on nanoindentation measurement depend on, for example, the amount of the surrounding phases in the microstructure, and/or their proximity to the indented phase. 

In these alloys, FeB and Fe_2_B phases are found in different combinations both with harder phases, e.g., V_3_B_4_, CrB_2_, CrB_4_, Cr_3_B_4_, B, and B_4_C, and/or softer, e.g., γ-Fe and graphite, as surrounding phases. Herein, in an attempt to minimize the influence of the surrounding phases, the diameters (d) of the phases measured were carefully chosen at d > 10 μm, and distance of about one-indent diameter was kept from the phase border during testing. Hence, for the alloys used in this study, the extrinsic effects can be considered minimized, although it cannot be claimed that they are eliminated entirely. On the contrary, the indentation hardness and modulus of Fe_2_B boride formed in the 82 Fe–9 B–9 Mn alloy and FeB in the 39.7 Fe–33 B–27.3 C alloy are probably underestimated due the extrinsic influences of the softer γ-Fe matrix and Fe_2_B phase, respectively. 

**Figure 7:**
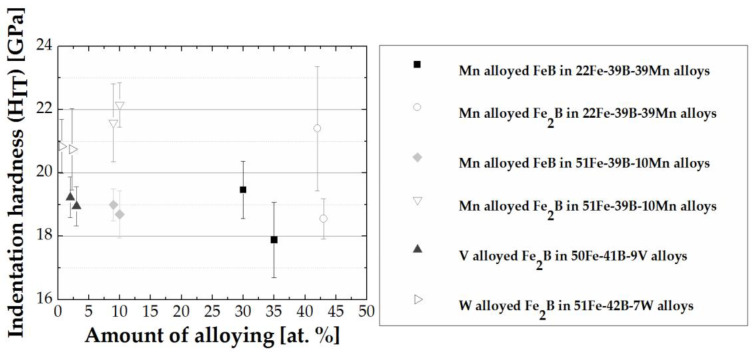
Amount of alloying (at.%) vs. indentation hardness (H_IT_) (GPa).

The relationship between amount of alloying and indentation hardness of alloys grouped by equal chemical composition is given in Figure 7. A trend of lower hardness at higher alloying content is observed, with exception to the Fe_2_B phase in alloy group 51 Fe–39 B–10 Mn. The differences between alloys in a group are within standard deviation at lower amounts of alloying content, and the difference within alloy group increases with increases in alloying content. The anomalous behavior in 51 Fe–39 B–10 Mn alloys cannot be claimed as inherent to the material and can be an outcome of the nanoindentation measurement process.

In general, the hardness of materials is primarily related to the mobility of dislocations [55]. However, at a more fundamental level, intrinsic hardness modification has been successfully linked to electronic structure effects induced by the alloying elements [56]. In the literature, the understanding of the underlying mechanism of electronic mechanical properties modification imposes a challenge, and its investigation is beyond the scope of this study. Herein, the measured indentation hardness is linked to a parameter called valence electron concentration (VEC) that has been used as an indicator for electronic modification of mechanical properties. The aim is to assess any possible relation of the VEC parameter to the nanoindentation hardness measured in this study.

**Figure 8:**
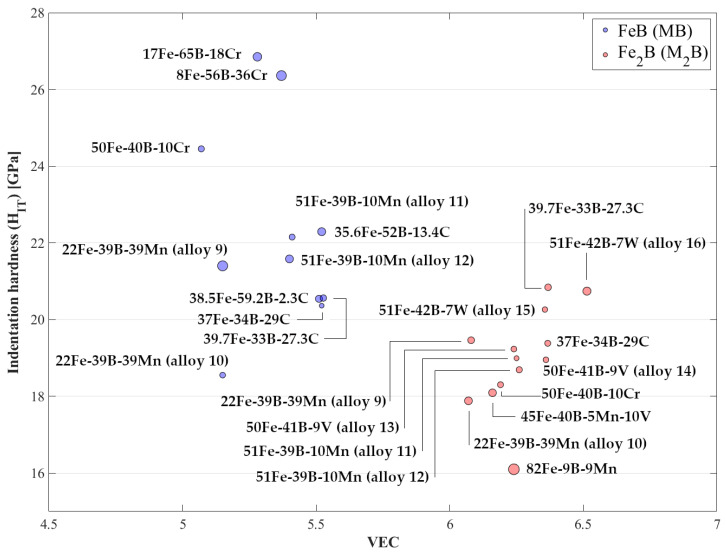
Valence electron concentration vs. indentation hardness (H_IT_) map for FeB and Fe_2_B borides.

The valence electron concentration (VEC) is defined as the number of valence electrons per formula unit [57]. VEC is calculated as given in Ge et al. [58]. The following valence electron numbers are used: 3 (B), 8 (Fe), 4 (C), 6 (Cr), 7 (Mn), 5 (V), and 6 (W), in determining VEC [59]. The calculation was done using the borides chemical composition as given in Table 1. VEC and indentation hardness for the Fe_2_B and FeB are given in the map in Figure 8. The map shows strong partitioning between the different phases, i.e., FeB and Fe_2_B. The typical values for single element alloyed FeB in these alloys are between 5.07 VEC to maximum 5.53 VEC, and for the Fe_2_B are in between mininum 6.07 VEC and maximum 6.36 VEC. Comparing FeB and Fe_2_B, the indentation hardness shows a decreasing trend with increasing VEC. This is due to the fact that with increasing VEC, the ‘metallic’ character of the materials increases, which could result in easier slip on a given slip system or the activation of more systems.

In the case of the Fe_2_B phase formed in the quaternary 45 Fe–40 B–5 Mn–10 V alloy, the map shows that the synergistic effect of Mn and V (alloying content of Mn = 5 at.% and V = 3 at.%), results in VEC = 6.16, and the measured indentation hardness is 18.1 ± 1.2 GPa. Although the boride structure type is the hardness determinant and in comparison to only manganese alloyed Fe_2_B, there is unclear disposition (because of the wider range of measured hardness data), the influences caused by the different elements are viable; since, in the presence of both manganese and vanadium, the indentation hardness is lower compared to only vanadium alloyed borides, i.e., 19.2 ± 0.6 GPa (alloy 13), 19.0 ± 0.6 GPa (alloy 14). 

Further, even though some clustering of hardness values at the same VEC is present, a strong tendency is not observable. Among the groups of alloys mentioned above, i.e., alloys with the same alloying element and heat-treated at the same temperature, i.e., FeB in 17 Fe–65 B–18 Cr and 50 Fe–40 B–10 Cr, FeB in manganese alloys annealed at 873 K, and FeB in manganese alloys annealed at 1223 K, as well as alloy groups that have the same chemical composition and/or phase composition, i.e., FeB in 51 Fe–39 B–10 Mn, Fe_2_B in 50 Fe–41 B–9 V, and Fe_2_B in 22 Fe–39 B–39 Mn, Fe_2_B in manganese alloys annealed at 1223 K the VEC vs. H_IT_ map shows that primarily lower hardness is observed at lower VEC values. However, this tendency is partially substantiated, i.e., it is not observed for the rest of the alloy groups where lower hardness is present at higher VEC values. For the C alloyed FeB annealed at 1173 K, alloy group 22 Fe–39 B–39 Mn, and manganese alloys annealed at 873 K, no relation can be discerned. The empirical relations between indentation hardness and VEC parameter obtained in this study can be used as orientation points for hardness estimation of similar borides. 

### 3.3. Indentation Size Effect

**Figure 9:**
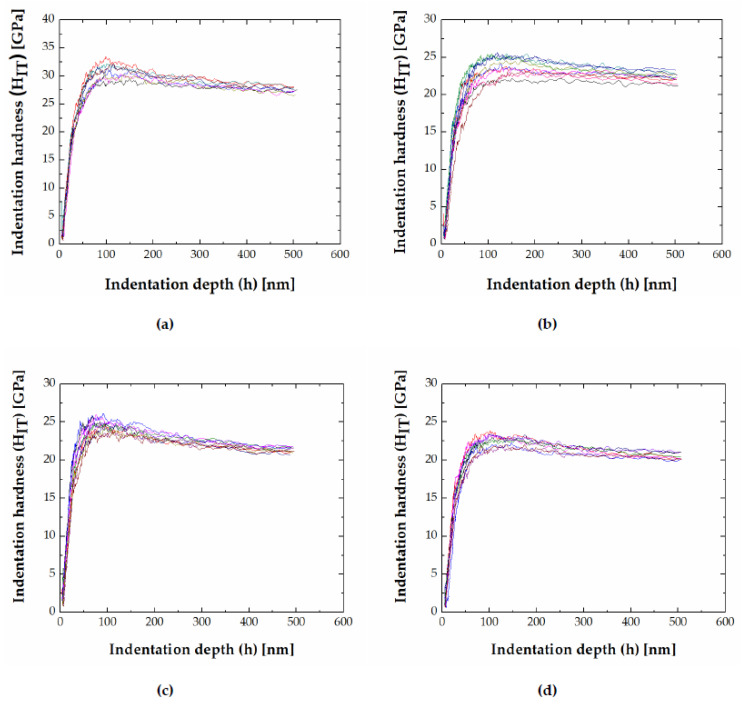
Indentation hardness vs. depth (**a**) FeB in 50 Fe–40 B–10 Cr, (**b**) MB in 51 Fe–39 B–10 Mn, (**c**) Fe_2_B in 39.7 Fe–33 B–27.3 C, (**d**) Fe_2_B in 45 Fe–40 B–5 Mn–10 V.

**Figure 10:**
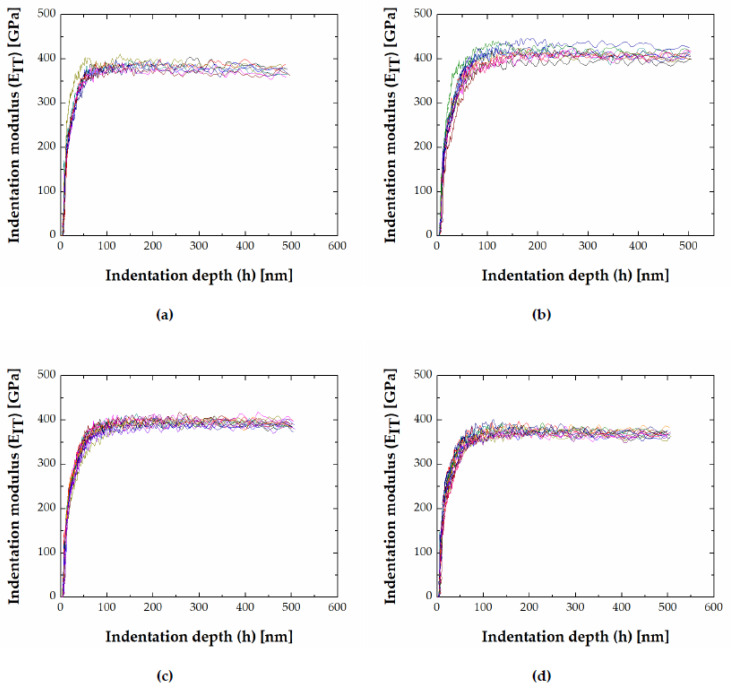
Indentation modulus vs. depth (**a**) FeB in 39.7 Fe–33 B–27.3 C, (**b**) MB in 51 Fe–39 B–10 Mn, (**c**) Fe_2_B in 50 Fe–40 B–10 Cr, (**d**) Fe_2_B in 50 Fe–41 B–9 V (alloy 14).

In the investigated alloys, an indentation size effect (decreasing hardness with indentation depth) in FeB and Fe_2_B was observed. Indentation hardness (H_IT_) and indentation modulus (E_IT_) for the FeB and Fe_2_B phase as a function of indentation depth (h) for chosen alloys are visualized in Figure 9 and Figure 10 (the different colored curves represent an individual indentation measurement). 

Indentation hardness vs. depth plots show that after reaching peak value (at approximately 50–100 nm), the indentation hardness decreases monotonically with increasing depth (Figure 9). The ISE for the FeB phase is most pronounced in the 50 Fe–40 B–10 Cr alloy, and for the Fe_2_B phase, in alloy 50 Fe–41 B–9 V (alloy 14). The ISE is more pronounced for Fe_2_B phase compared to FeB phase (Figure 9).

Indentation modulus vs. depth plots show an initial increase to a maximum value followed by a subsequent decrease up to a constant value (Figure 10). The constant modulus indicates that intrinsic materials’ properties were measured.

**Figure 11:**
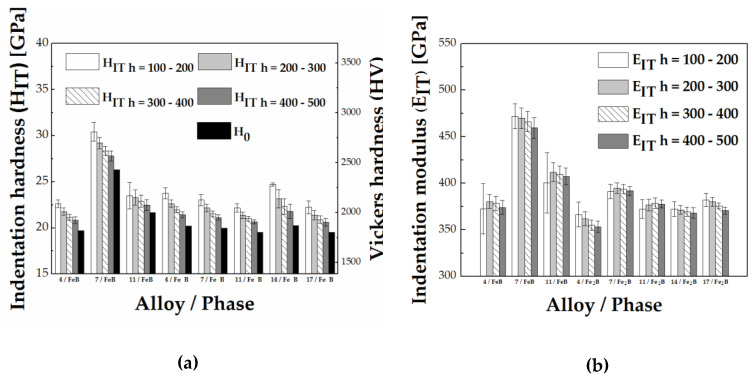
(**a**) Indentation hardness, H_IT_, as average values in indentation depth intervals of h = 100–200, 200–300, 300–400, 400–500, and true hardness H_0_. (**b**) Indentation modulus, E_IT_, as average values in indentation depth intervals of h = 100–200, 200–300, 300–400, 400–500.

The change of indentation hardness (H_IT_) and indentation modulus (E_IT_) at different depth intervals (h = 100–200 nm, h = 200–300 nm, h = 300–400 nm, h = 400–500 nm) for the FeB and Fe_2_B phase is visualized in Figure 11.

**Figure 12:**
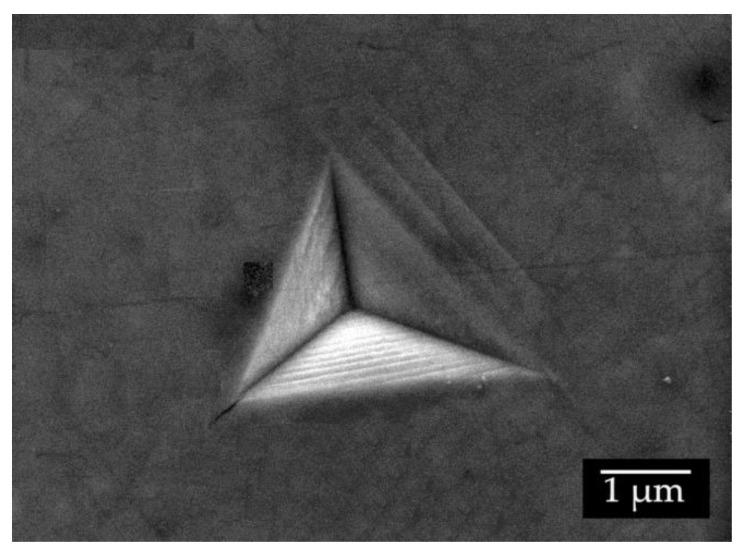
Steps and slip lines near indent in the FeB phase in the microstructure of alloy 50 Fe–40 B–10 Cr.

Indentation size effect is a well-known phenomenon in indentation testing and various mechanisms have been identified as responsible for ISEs, such as dislocations, cracking, phase transformations, surface effects, etc. [60]. In the case of crystalline metals, the dislocation-based mechanisms are identified as the dominant underlying mechanisms of ISE [60]. The Nix and Gao model [61] is an established model used to estimate hardness based on the dislocation-based behavior as a prevailing mechanism influencing ISEs. Observed slip lines and linear details (steps) in the FeB phase in alloys 50 Fe–40 B–10 Cr (Figure 12) signify a dislocation-based deformation behavior. Dislocation-based deformation for the Fe_2_B was also reported by Lentz et al. [29].

Thus, the Nix and Gao model has been applied to calculate the hardness at infinite indentation depth or true hardness (H_0_) of both FeB and Fe_2_B phases. The calculated true hardness (H_0_) based on the Nix–Gao model is given in Table 4.

**Figure 13:**
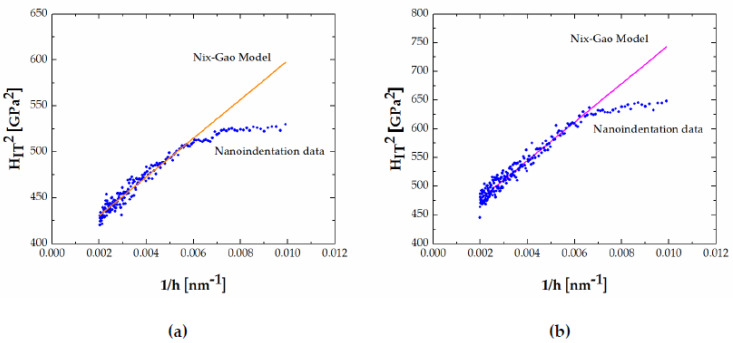
H_IT_^2^ vs. h^−1^ plot: (**a**) FeB in 39.7 Fe–33 B–27.3 C, and (**b**) Fe_2_B in 50 Fe–41 B–9 V (alloy 14).

The model was fitted to data for indentation depth >100 nm (Figure 13). The H_IT_^2^ vs. h^−1^ plot showed good fit of the Nix–Gao model for both FeB and Fe_2_B phases. However, as one can see, the plots for FeB and Fe_2_B phase show linear behavior at larger depths, but the linearity does not extend to smaller depths (h < approximately 150 nm). In the literature, this behavior has been interpreted as bilinear and considered as an indicator of change in the prevailing ISE mechanism at smaller depths [60,62].

## 4. Conclusions

In this study, the influence of the third element dissolved in FeB and Fe_2_B phases formed in different Fe-B-X (X = C, Cr, Mn, V, W, Mn + V) systems has been characterized by nanoindentation. The results of this study can be outlined as follows:

(1) The determined indentation hardness under the influence of different amounts and type of alloying elements showed the highest hardness of FeB formed in Fe-B-X (X = C, Cr, Mn) systems in the presence of Cr as an alloying element with a hardness value of H_IT_ = 26.9 ± 1.4 GPa. The highest hardness in the Fe_2_B was measured in the presence of W additions H_IT_ = 20.8 ± 0.9 GPa. The lowest hardness in both alloys was determined in Mn alloyed FeB and Fe_2_B.
(2) The highest hardness for FeB boride was measured at VEC = 5.28, and for the Fe_2_B boride, at VEC = 6.368. Comparison between FeB and Fe_2_B showed, overall, that the indentation hardness decreases with increasing VEC, which is associated with the increase of the ‘metallic’ character of the materials and easier slip on a given slip system or the activation of more systems.
(3) The determined indentation modulus under the influence of different amounts and type of alloying elements showed that Cr alloyed FeB and Fe_2_B are stiffest, E_IT_ = 485.5 ± 22.3 GPa and E_IT_ = 391.7 ± 10.9 GPa, respectively. The lowest modulus in the case of FeB formed in Fe-B-X (X = C, Cr, Mn) was measured in the presence of C as an alloying element, while for Fe_2_B, in Mn alloyed Fe_2_B.
(4) The indentation size effect was observed in both FeB and Fe_2_B phases and the hardness decreased with an increase in the indentation depth. The nanoindentation data has been successfully fitted to a dislocation-based model for determining the real hardness, H_0_.

## Figures and Tables

**Table 1:** Alloys’ chemical composition, annealing conditions, identified phases, and borides chemical composition.

Alloy	Alloy Composition (at.%)	Annealing Conditions T (K)/time (h)	Phase Composition	Fe_2_B (M_2_B) Chemical Composition (at.%)	FeB (MB) Chemical Composition (at.%)
1	38.5 Fe–59.2 B–2.3 C	1173/1000	FeB + B_4_C + B	-	48.96 Fe, 50 B, 1.04 C
2	34.6 Fe–52 B–13.4 C	1173/1000	FeB + B_4_C + graphite	-	47.3 Fe, 50 B, 2.7 C
3	37 Fe–34 B–29 C	873/1000	Fe_2_B + FeB + graphite	30.01 Fe, 66.67B, 3.32C	47.92 Fe, 50 B, 2.08 C
4	39.7 Fe–33 B–27.3 C	1173/1000	Fe_2_B + FeB + graphite	31.06 Fe, 66.67B, 2.27C	47.31 Fe, 50 B, 2.69 C
5	17 Fe–65 B–18 Cr	1353/1848	FeB + CrB_4_ + CrB_2_	-	39 Fe, 50 B, 11 Cr
6	8 Fe–56 B–36 Cr	873/2300	FeB + Cr_3_B_4_	-	37 Fe, 51 B, 12 Cr
7	50 Fe–40 B–10 Cr	1353/1848	Fe_2_B + FeB	59 Fe, 33 B, 8 Cr	35 Fe, 49 B, 16 Cr
8	82 Fe–9 B–9 Mn	1223/1440	Fe_2_B + γFe	56 Fe, 33 B, 11 Mn	-
9	22 Fe–39 B–39 Mn	873/2160	M_2_B + MB	28 Fe, 30 B, 42 Mn	19 Fe, 51 B, 30 Mn
10	22 Fe–39 B–39 Mn	1223/1440	M_2_B + MB	27 Fe, 30 B, 43 Mn	15 Fe, 50 B, 35 Mn
11	51 Fe–39 B–10 Mn	873/2160	M_2_B + MB	57 Fe, 33 B, 10 Mn	41 Fe, 50 B, 9 Mn
12	51 Fe–39 B–10 Mn	1223/1440	M_2_B +MB	58 Fe, 33 B, 9 Mn	40 Fe, 50 B, 10 Mn
13	50 Fe–41 B–9 V	1353/1440	Fe_2_B + V_3_B_4_	64 Fe, 34 B, 2 V	-
14	50 Fe–41 B–9 V	903/4560	Fe_2_B + FeB + V_3_B_4_	66 Fe, 31 B, 3 V	39.2 Fe, 51 B, 9.8 V
15	51 Fe–42 B–7 W	1323/2000	Fe_2_B + FeB + W_2_FeB_2_	67 Fe, 32.4 B, 0.6 W	41 Fe, 50 B, 1 W
16	51 Fe–42 B–7 W	950/4224	Fe_2_B + FeB + W_2_FeB_2_	68.89 Fe, 28.81 B, 2.3 W	47.86 Fe, 49.54 B, 2.6 W
17	45 Fe–40 B–5 Mn–10 V	900/2040	M_2_B + MB + V_3_B_4_	58 Fe, 34 B, 5 Mn, 3 V	36 Fe, 50 B, 5 Mn, 9 V

(-) the phase is not present in the alloy.

**Table 2:** The phase fraction of the alloys.

Alloy	Phase Fraction (vol. %)
1	FeB: 77.28, B_4_C: 11.44, B: 11.28 **
2	FeB: 54.25, B_4_C: 6.32, graphite: 39.43 **
3	Fe_2_B: 20,02, FeB: 52,09, graphite: 27,89 **
4	Fe_2_B: 48.64, FeB: 23.42, graphite: 27.94 **
5	FeB: 58.20, CrB_4_: 21.56, CrB_2_: 20.24 **
6	FeB: 2.87, Cr_3_B_4_: 97.13 **
7	Fe_2_B: 71.35, FeB: 28.65 **
8	Fe_2_B: 27, γ-Fe:73 *
9	M_2_B: 66, MB: 34 *
10	M_2_B: 67, MB: 33 *
11	M_2_B: 68.6, MB: 31.4 *
12	M_2_B: 68.3, MB: 31.7 *
13	Fe_2_B: 76.7, V_3_B_4_: 23.3 *
14	Fe_2_B: 63.8, FeB: 21.6, V_3_B_4_: 14.6 *
15	Fe_2_B: 25.65, FeB: 48.58, W_2_FeB_2_: 25.77 **
16	Fe_2_B: 37.78, FeB: 44.85, W_2_FeB_2_: 17.37 **
17	M_2_B: 60.02, MB: 21.64, V_3_B_4_: 18.34 **

(*) calculated using the lever rule, (**) determined using image analysis

**Table 3:** Indentation hardness (H_IT_) and indentation modulus (E_IT_) in GPa for FeB and Fe_2_B phase

Alloy	Alloy’s Chemical Composition	H_IT_ FeB	E_IT_ FeB	H_IT_ Fe_2_B	E_IT_ Fe_2_B
1	38.5 Fe–59.2 B–2.3 C	20.5 ± 1.0	370.3 ± 20.6	-	-
2	34.6 Fe–52 B–13.4 C	22.3 ± 1.3	358.5 ± 30.0	-	-
3	37 Fe–34 B–29 C	20.4 ± 0.5	315.6 ± 32.8	19.4 ± 0.7	315.7 ± 21.7
4	39.7 Fe–33 B–27.3 C	20.6 ± 0.8	375.8 ± 22.2	20.3 ± 0.6	350.4 ± 14.3
5	17 Fe–65 B–18 Cr	26.9 ± 1.4	485.5 ± 22.3	-	-
6	8 Fe–56 B–36 Cr	26.4 ± 1.9	410.1 ± 27.3	-	-
7	50 Fe–40 B–10 Cr	24.5 ± 0.7	437.4 ± 17.1	18.3 ± 0.7	391.7 ± 10.9
8	82 Fe–9 B–9 Mn	-	-	16.1 ± 2.2	297.5 ± 24.6
9	22 Fe–39 B–39 Mn	21.4 ± 2.0	374.3 ± 36.6	19.5 ± 0.9	354.4 ± 11.2
10	22 Fe–39 B–39 Mn	18.6 ± 0.6	375.0 ± 11.2	17.9 ± 1.2	315.1 ± 35.9
11	51 Fe–39 B–10 Mn	22.2 ± 0.7	385.5 ± 25.4	19.0 ± 0.5	375.2 ± 8.8
12	51 Fe–39 B–10 Mn	21.6 ± 1.2	400.5 ± 15.0	18.7 ± 0.7	367.9 ± 8.9
13	50 Fe–41 B–9 V	-	-	19.2 ± 0.6	354.5 ± 9.5
14	50 Fe–41 B–9 V	-	-	19.0 ± 0.6	364.1 ± 8.0
15	51 Fe–42 B–7 W	-	-	20.8 ± 0.9	383.1 ± 8.7
16	51 Fe–42 B–7 W	-	-	20.7 ± 1.3	377.8 ± 8.7
17	45 Fe–40 B–5 Mn–10 V	-	-	18.1 ± 1.2	367.4 ± 18.7

(-) phase is not present or not measured due to small diameter.

**Table 4:** Real hardness, H_0_ (GPa), for Fe_2_B and FeB phases.

Alloy	Chemical Composition	H_0_-Fe_2_B (M_2_B)	H_0_-FeB (MB)
4	39.7 Fe–33 B–27.3 C	20.19	19.70
7	50 Fe–40 B–10 Cr	19.98	26.31
11	51 Fe–39 B–10 Mn	19.54	21.67
14	50 Fe–41 B–9 V	20.26	-
17	45 Fe–40 B–5 Mn–10 V	19.54	-
